# Influence of beat-to-beat blood pressure variability on vascular elasticity in hypertensive population

**DOI:** 10.1038/s41598-017-08640-4

**Published:** 2017-08-21

**Authors:** Yufa Xia, Xin Liu, Dan Wu, Huahua Xiong, Lijie Ren, Lin Xu, Wanqing Wu, Heye Zhang

**Affiliations:** 1 Shenzhen institutes of advanced technology, Chinese Academy of Sciences, Shenzhen, China; 2 Department of Ultrasonography, The Second People’s Hospital of Shenzhen, Shenzhen, China; 3 Shenzhen College of Advanced Technology, University of Chinese Academy of Sciences, Shenzhen, China; 4 Department of Neurology, The Second People’s Hospital of Shenzhen, Shenzhen, China; 5 Department of Cardiology, Genaral hospital of Guanhzhou Military command of PLA, Guangzhou, China

## Abstract

Whether elevated beat-to-beat blood pressure variability (BPV) has an influence on vascular elasticity is confounded and poorly understood. This study hypothesized that the increased BPV could have an adverse effect on the vascular elasticity, as estimated by total arterial compliance (TAC), independent of blood pressure (BP) values. Beat-to-beat BP and TAC were measured in 81 hypertensive patients (experimental population) and in 80 normal adults (control population). Beat-to-beat BPV was assessed by standard deviation (SD), average real variability (ARV), residual standard deviation (RSD) and variation independent of mean (VIM). In experimental population, systolic BPV (SBPV) showed a significant correlation with TAC (SD, r = −0.326, p < 0.001; ARV, r = −0.277, p = 0.003; RSD, r = −0.382, p < 0.001; VIM, r = −0.274, p = 0.003); similarly, SD, RSD and VIM of diastolic BP (DBP) also showed explicit correlation with TAC (r = −0.255, p = 0.006; r = −0.289, p = 0.002; r = −0.219, p = 0.019; respectively). However, in the control population, neither SBPV nor diastolic BPV (DBPV) showed a significant correlation with TAC. Furthermore, in the experimental population, VIM of systolic BP (SBP) was also a determinant of TAC (β = −0.100, p = 0.040) independent of average SBP, DBP, age and body mass index. In conclusion, these data imply that beat-to-beat BPV, especially SBPV, shows an independent correlation with vascular elasticity in hypertensive population.

## Introduction

Cardiovascular disease (CVD) remains the number one cause of death globally; almost 17.5 million people died from CVD in 2012, accounting for 31% of all global deaths, and the number may reach up to 23.3 million by 2030^1^. Although high blood pressure (BP) values have been considered to evoke the adverse influence of the CVD, increasing evidence shows that the increased BP variability (BPV) is also associated with the development of the CVD^2^. Short-term BPV, which includes 24-h BPV and beat-to-beat BPV, has been confirmed as a risk factor of CVD events^3–6^. Upon most occasions, the standard deviation (SD) of the BP measurements is used as the clinical evaluation of short-term BPV^2^. However, SD is limited to estimate the BPV in multivariate module, because SD only reflects the global fluctuation of the BP values around the mean level and does not take the order of BP measurements into account^7–9^. To overcome these drawbacks of the SD, some novel parameters have been proposed to estimate BPV. These novel parameters include average real variability (ARV), residual standard deviation (RSD) and variation independent of mean (VIM). ARV takes the sequence of BP measurements into account^7^. RSD can reflect the underlying trend between BP values and time^8^. VIM is transformed by the SD, and it can exclude the impact of mean BP levels^9^.

The vascular damage increases CVD risk, and it occurs before any clinical feature of the CVD^10^. Vascular damage accompanies elevated arterial stiffness and reduced arterial elasticity. Total arterial compliance (TAC) is an evaluation of the arterial compliance in the entire arterial tree^11^, and it reflects the elastic property of the vascular system^12–14^. Furthermore, TAC plays an important role in the biomechanics and homeostasis^10^, and it is the main determinant of cardiac afterload, left ventricular function and arterio-ventricular coupling^15–19^. The decreased TAC represents the reduction of the vascular elasticity. And reduced TAC is considered as the optimal clinical index of damaged pulsatile arterial function and signals the early vascular damage^20^. Recently, some studies have manifested that 24-h BPV shows significant association with vascular damage^6, 21–23^. Although beat-to-beat BPV is considered more precise than 24-h BPV to evaluate the short-term BPV^5, 6^, the association between beat-to-beat BPV and vascular damage is non-significant^6^.

The aim of this study was to explore the influence of beat-to-beat BPV on vascular elasticity, estimated by TAC, in hypertensive population. In this study, we measured the beat-to-beat BP parameters and TAC by noninvasive method in hypertensive population and normal population. We used the hypertensive subjects as experimental group and used the normal subjects as a control group.

## Methods

### Study population

The study protocol was designed according to the principles of the Declaration of Helsinki and then was approved by the Clinical Ethics Committee of the Second People’s Hospital of Shenzhen, China. The experimental data were available. All subjects volunteered to participate in this study, and the written informed consent forms were obtained from each subject before the experiment. And all of data were available. The study population included two separate populations, an “experimental population” of hypertensive patients and a “control population” of normal adults. The latter data analysis was utilized to confirm the findings obtained in the experimental population. The experimental population consisted of 81 hypertensive patients with an age range of 29–75 years (42 males, 51.85%). All of the hypertensive patients fulfilled the following inclusion criteria: (a) SBP larger than 140 mmHg or DBP larger than 90 mmHg; (b) no history of diabetes mellitus, dyslipidemia; (c) no clinical or laboratory evidence of heart failure, coronary artery disease, cerebrovascular disease. With the objective of confirming the findings of the experimental population, we included another completely independent population of 80 normal adults (control population, 44 males, 55.0%, age range 28 to 75 years). All of control subjects met following inclusion criteria: (a) no history of hypertension, and SBP smaller than 140 mmHg and DBP smaller than 90 mmHg; (b) no history of diabetes mellitus, dyslipidemia; (c) no clinical or laboratory evidence of heart failure, coronary artery disease, cerebrovascular disease; (d) no history of drug abuse. Both the experimental subjects and the control subjects were recruited in the Second People’s Hospital of Shenzhen, Shenzhen, China from April to December 2014.

### Cardiovascular signals measurements

All of the subjects underwent clinical examination in the two populations. In both of the populations, cardiovascular signals of the patients were recorded by Finometer® MIDI (Model II, Finapres Medical Systems B.V., The Netherlands) and were stored in a personal computer with a BeatScope® Easy software (Finapres Medical Systems B.V., The Netherlands). And these cardiovascular signals mainly included beat-to-beat BP, ECG and stroke volume. In the Finometer® MIDI, the beat-to-beat BP is recorded by volume-clamp method, which is kept the arterial diameter constant with a cuff wraps around the finger^24^. And the correct arterial diameter is maintained by Physiocal algorithm^25^. While, the Modelflow method is utilized to measure stroke volume^24, 26^. In the Modelflow method, a non-linear three-element model, representing three major characteristics of aortic input impedance, is utilized to calculate the stroke volume precisely^26^. All subjects were instructed to lie in the supine position on a single bed quietly when the cardiovascular signals were measured. The procedure of cardiovascular signals measurement lasted ten minutes. Moreover, body weight and height were measured with the subjects without shoes and in light clothing. And body mass index (BMI) was estimated by the ratio of weight (kg) to the height square (m^2^). Caffeine, smoking and alcohol use was also recorded. All the subjects were asked to refrain from caffeine, cigarette, alcohol, or severe physical activities for 3 ~ 4 hours prior to the experiment.

### Beat-to-beat blood pressure variability assessment

Because SD of the BP values had some disadvantages, such as only exhibiting the distribution of BP measurements around the mean levels and not accounting for the time sequence of BP recordings, it was limited to assess the BPV only using the SD of BP values. To overcome these limitations, we added the ARV, RSD and VIM to evaluate the beat-to-beat BPV besides SD. These parameters were calculated using the following formulas:

1 $$SD=\sqrt{\frac{1}{n-1}\sum _{i=1}^{n}{({X}_{i}-\overline{X})}^{2}}$$

2 $$ARV=\frac{1}{n-1}\cdot \sum _{i=1}^{n}|{X}_{i+1}-{X}_{i}|$$

3 $$RSD=\sqrt{\frac{1}{n-2}\sum _{(i=1)}^{n}{({X}_{i}-\hat{{X}_{i}})}^{2}}$$

4 $$VIM=k\cdot SD/{\overline{X}}^{m}$$

where × _1_, × _2_, …, X_n_ denoted a set of BP measurement values; $\overline{X}$ was the mean value of the set of BP measurement values; ${\widehat{X}}_{1}$, ${\widehat{X}}_{2}$, …, ${\widehat{X}}_{n}$ were the fitted values from a linear regression of BP against time; k and m were obtained from a fitting curve of the form *y* = *kx* ^*p*^ through a plot of SD BP (y-axis) against mean BP (x-axis).

### Total arterial compliance measurements

It is lack of a ‘gold standard’ to assess TAC. Some noninvasive approaches have been used to estimate TAC, and they derived from the Windkessel model^15, 27, 28^. Chemla *et al*.^15^ studied the estimation of TAC in human at rest, and they demonstrated that the stroke volume/pulse-pressure method (SV/PP) was a reliable method to assess TAC. Moreover, Haluska *et al*.^29^ compared the pulse-pressure method (PPM), area method (AM) and SV/PP to evaluate TAC; and they found that each of the approaches showed significant correlation with each other; and SV/PP and PPM were more robust than the AM. In addition, Simone *et al*.^16^ used SV/PP to evaluate TAC, and they demonstrated that SV/PP was a predictor of cardiovascular morbid events independent of age and LV mass index in arterial hypertension; Wohlfahrt *et al*.^17^ also used SV/PP to estimate TAC, and they found that chronic changes TAC were differentially correlated with age-related LV stiffening and chamber remodelling. In this study, SV/PP was utilized to assess the TAC. We measured stroke volume, beat-to-beat SBP and beat-to-beat DBP, and the pulse-pressure (PP) was the beat-to-beat SBP minus the beat-to-beat DBP.

### Statistical analysis

The Statistical Package for the Social Sciences (SPSS) 19.0 (SPSS Inc., Chicago, Illinois, USA) was used for statistical analysis. Continuous variables were tested to detect substantial deviations from normality by computing the Kolmogorov-Smirnov Z, and the assumption of satisfactory normal distribution was met for all of the examined variables. Descriptive statistics were presented as mean ± SD. Independent-sample T test was used to test the difference of parameters in the two groups. Bivariate correlation analysis was used to investigate the bivariate associations. Multivariate linear regression analysis (stepwise criteria: Probability of F-to-enter ≥ 0.050, probability of F-to-remove ≥ 0.100) were used to elucidate the independent determinants of TAC. P < 0.05 was considered statistically significant.

## Results

### Demographic and clinical characteristics data

Demographic data of the experimental population and control population was listed in Table 1. In the experimental population, the number of the subjects was 81, including 42 males (51.85%); their age ranged from 29–75 years, averaging (Mean ± SD) 56.7 ± 10.1 years. In the control population, it consisted of 80 subjects, and included 44 males (55.0%); the range of age was from 28 to 75 years, and the mean ( ± SD) was 49.5 ± 11.5 years. Moreover, in two populations, their height, weight and BMI were also recorded in Table 1. Furthermore, caffeine, smoking and alcohol use was also shown in Table 1. In independent-sample T test, these parameters were not significantly different between hypertensive population and normal population (p ≥ 0.413).

**Table 1:** Clinical characteristics of hypertensive subjects and normal subjects.

variable	Experimental population	Control population	P
	(Mean ± SD, N = 81)	(Mean ± SD, N = 80)	
Age (year)	56.7 ± 10.1	49.5 ± 11.5	0.413
Male (%)	42 (51.85%)	44 (55.0%)	0.849
Height (cm)	165.7 ± 7.8	165.9 ± 7.5	0.861
Weight (kg)	69.0 ± 10.8	63.8 ± 11.2	0.422
BMI (kg/m^2^)	25.0 ± 2.8	23.1 ± 3.2	0.517
Smokers (%)	17 (20.99%)	15(18.75%)	0.730
Alcohol (%)	5(6.17%)	4(5.0%)	0.813
Coffee (%)	3 (3.70%)	3(3.75%)	0.917

SD, standard deviation; BMI, body mass index.

Table 2 showed the beat-to-beat SBP, beat-to-beat DBP, beat-to-beat SBP variability (SBPV), beat-to-beat DBP variability (DBPV), pulse pressure (PP), stroke volume, HR and TAC values in hypertensive population and normal population. In independent-sample T test, SBP, PP and all the indices of SBPV in hypertensive population were larger than the values in normal population (p ≤ 0.008); however, the DBP and all the indices of DBPV in hypertensive population were not different from the values in normal population significantly (p ≥ 0.096). Similarly, HR parameters in hypertensive population were also not different from the values in normal population significantly (p ≥ 0.184). Furthermore, the stroke volume and TAC in hypertensive population were smaller than the values in normal population remarkably (p ≤ 0.041).

**Table 2:** BP parameters, HR, pulse pressure, stroke volume and TAC of 81 hypertensive subjects and 80 normal subjects.

variable	Experimental population	Control population	P
	(Mean ± SD, N = 81)	(Mean ± SD, N = 80)	
SBP (mmHg)	143.6 ± 20.8	121.4 ± 11.4	<0.001**
SBP_SD (mmHg)	7.46 ± 2.51	5.76 ± 1.58	0.003**
SBP_ARV (mmHg)	2.53 ± 1.03	1.87 ± 0.60	0.001**
SBP_RSD (mmHg)	6.87 ± 2.30	5.36 ± 1.44	0.008**
SBP_VIM (Unit)	7.45 ± 2.44	5.76 ± 1.57	0.006**
DBP (mmHg)	76.5 ± 9.9	67.9 ± 9.1	0.358
DBP_SD (mmHg)	4.28 ± 1.47	3.61 ± 1.16	0.296
DBP_ARV (mmHg)	1.37 ± 0.69	1.23 ± 0.47	0.096
DBP_RSD (mmHg)	4.06 ± 1.42	3.45 ± 1.10	0.436
DBP_VIM (Unit)	4.29 ± 1.47	3.62 ± 1.17	0.467
PP (mmHg)	66.0 ± 17.10	53.5 ± 7.8	<0.001**
StrV (mL)	76.7 ± 18.7	84.6 ± 17.6	0.041*
HR (beats/min)	70.8 ± 11.5	67.3 ± 10.4	0.395
HR_SD (beats/min)	70.6 ± 11.7	67.3 ± 10.4	0.184
TAC (mL/mmHg)	1.14 ± 0.38	1.59 ± 0.31	0.037*

SBP, systolic blood pressure; DBP, diastolic blood pressure; SD, standard deviation; ARV, average real variability; RSD, residual standard deviation; VIM, variation independent of mean; HR, heart rate; PP, pulse pressure; StrV, stroke volume; TAC, total arterial compliance. *p < 0.05; **P < 0.01.

### Bivariate correlation analysis

The Pearson’s correlations of TAC with anthropometric measures values, HR values, PP and stroke volume, were shown in Table 3. The anthropometric measures values mainly included gender, age, height, weight and BMI. Both in hypertensive subjects and in normal subjects, the gender, height, weight, BMI and stroke volume had significantly positive correlations with TAC, while the age and PP showed negative correlations with TAC (as showed in Table 3). However, correlations of TAC with HR and SD of HR were non-significant both in hypertensive population and in normal population.

**Table 3:** The Pearson’s correlations of TAC with anthropometric measures values in hypertensive population and normal population.

Variable	Experimental population		Control population	
	r	p	r	p
Gender (male/female)	0.291**	0.002	0.351**	0.001
Age (years)	−0.465**	<0.001	−0.260*	0.020
Height (m)	0.360**	<0.001	0.569**	<0.001
Weight (kg)	0.439**	<0.001	0.464**	<0.001
BMI (kg/m2)	0.310**	0.001	0.227*	0.043
HR (beats/min)	−0.151	0.106	−0.087	0.459
HR_SD (beats/min)	0.067	0.476	0.052	0.660
PP (mmHg)	−0.531**	<0.001	−0.276*	0.013
StrV (mL)	0.590**	<0.001	0.719**	<0.001

BMI, body mass index; HR, heart rate; TAC, total arterial compliance; PP, pulse pressure; StrV, stroke volume. *p < 0.05; **P < 0.01.

Table 4 showed the Pearson’s correlations of TAC with beat-to-beat BP and beat-to-beat BPV in hypertensive subjects and normal subjects. In hypertensive population, both SBP and DBP showed negative correlate with TAC significantly (r = −0.693, p < 0.001; r = −0.541, p < 0.001; respectively). The SD, ARV, RSD and VIM of SBP were also associated with TAC remarkably (r = −0.326, p < 0.001; r = −0.277, p = 0.003; r = −0.382, p < 0.001; r = −0.274, p = 0.003; respectively). Similarly, the SD, RSD and VIM of DBP were also correlated with TAC significantly (r = −0.255, p = 0.006; r = −0.289, p = 0.002; r = −0.219, p = 0.019; respectively). In normal population, correlations of TAC with SBP and DBP were significant (r = −0.438, p < 0.001; r = −0.313, p = 0.005; respectively); however, correlations between TAC and BPV were non-significant (p ≥ 0.330).

**Table 4:** The Pearson’s correlations of TAC with beat-to-beat BP and beat-to-beat BPV in hypertensive population and normal population.

Variable	Experimental population		Control population	
	r	p	r	p
SBP (mmHg)	−0.693**	<0.001	−0.438**	<0.001
SBP_SD (mmHg)	−0.326**	<0.001	−0.094	0.407
SBP_ARV (mmHg)	−0.277**	0.003	−0.088	0.435
SBP_RSD (mmHg)	−0.382**	<0.001	−0.081	0.476
SBP_VIM (Unit)	−0.274**	0.003	−0.067	0.556
DBP (mmHg)	−0.541**	<0.001	−0.313**	0.005
DBP_SD (mmHg)	−0.255**	0.006	0.070	0.539
DBP_ARV (mmHg)	−0.065	0.493	0.110	0.330
DBP_RSD (mmHg)	−0.289**	0.002	0.049	0.666
DBP_VIM (Unit)	−0.219*	0.019	0.091	0.422

SBP, systolic blood pressure; DBP, diastolic blood pressure; TAC, total arterial compliance; SD, standard deviation; ARV, average real variability; RSD, residual standard deviation; VIM, variation independent of mean. *p < 0.05; **P < 0.01.

### Stepwise multivariate linear regression analysis

**Figure 1:**
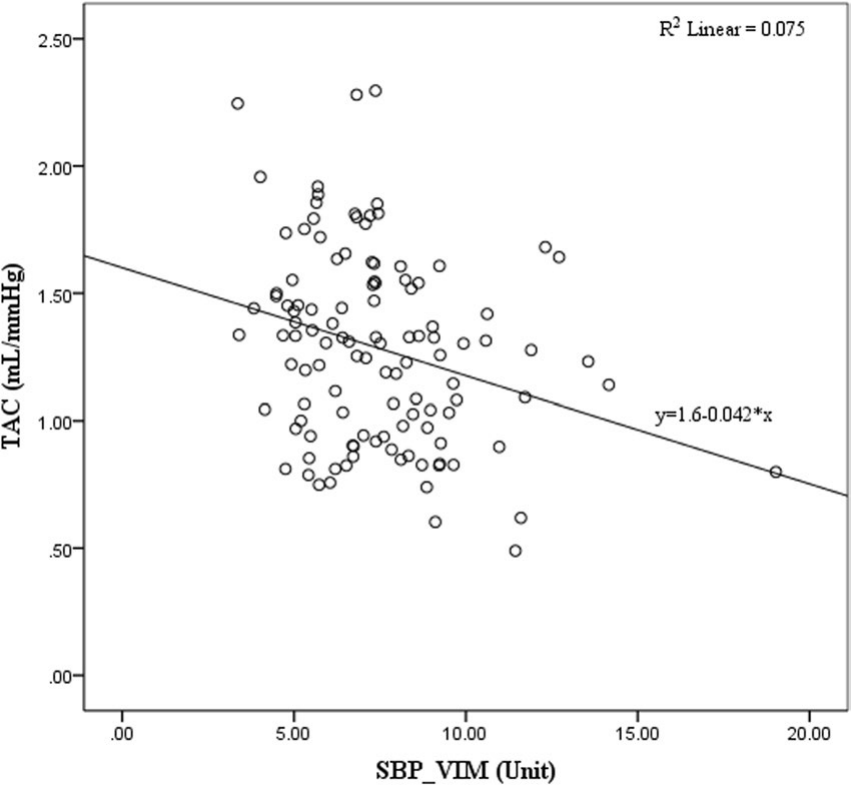
Linear relationship between TAC and VIM of SBP in hypertensive population. TAC, total arterial compliance; SBP, systolic blood pressure; VIM, variation independent of mean.

The independent relationships between BPV and TAC were examined in stepwise multiple linear regression models in hypertensive population. Before building the model, the test for multicollinearity among the tested variables was performed; and the variance inflation factor (VIF) was less than 5; that is to say, there is no multicollinearity among these tested variables. As Table 5 shown, VIM of beat-to-beat SBP was correlated with TAC (β = −0.100, p = 0.04) independent of SBP, DBP, age and BMI. Gender, Height, Weight, HR, SD of HR, SD of SBP, ARV of SBP, RSD of SBP and four indices of DBPV did not enter the final equation. The linear relationship between TAC and VIM of SBP was shown in Fig. 1.

**Table 5:** Independent Predictors of TAC in a Stepwise Multiple Linear Regression Analysis.

Variable	β	P value	R^2^
SBP	−0.125	0.071	0.775
Age	−0.502	<0.001	
DBP	−0.637	<0.001	
BMI	0.268	<0.001	
SBP_VIM	−0.100	0.040	

SBP, systolic blood pressure; DBP, diastolic blood pressure; TAC, total arterial compliance; BMI, body mass index; VIM, variation independent of mean.

## Discussion

In this study, our results provided unequivocal evidence of the association between vascular elasticity, as evaluated by TAC, and different parameters of beat-to-beat BPV, especially the beat-to-beat SBPV, in hypertensive population. Whereas, the correlation of vascular elasticity with beat-to-beat BPV was not significant in normal population. The evidence suggested that the elevated beat-to-beat BPV had an influence on the vascular elasticity and marked the early vascular damage in hypertensive patients.

The results of our study have some potential clinical implications. Our results showed that the correlation between reduced TAC and elevated beat-to-beat BPV, especially the beat-to-beat SBPV, was significant independent of average BP in hypertensive population. For hypertensive patients, although the reduction in average BP by treatment is important, the decreasing BPV may be also important for preventing cardiovascular morbidity and mortality. Moreover, the elevated BPV may be also an underlying mark of premature vascular lesions.

Although the level of high BP values had been confirmed as an important predictor of target-organ damage, which mainly included vascular, cardio, renal organ damage, over the past years^22^; the increased BPV values might be a new determinant of the development target-organ damage, especially vascular damage. BPV was regarded as an intricate interaction between intrinsic cardiovascular control mechanism and external environmental stimuli. The beat-to-beat BPV mainly reflected the influences of central sympathetic drive, arterial or cardiopulmonary reflex, humoral, rheological, behavioural and emotional factors, activity and sleep^5^. Few previous studies investigated the target-organ damage correlated with beat-to-beat BPV. Parati *et al*.^30^ measured 24-hour beat-to-beat BP in intra-arterial method and assessed target-organ damage in 108 untreated hypertensive subjects; they used SD of beat-to-beat BP to estimate the beat-to-beat BPV; and they manifested that the severity of target-organ damage increased with the elevated mean BP and BPV in nearly any level of the 24-hour beat-to-beat BP. Veerman *et al*.^31^ measured 20 minutes beat-to-beat BP in 33 untreated hypertensive subjects by Finapres Model 5; and they measured the left ventricular mass index (LVMI) and urinary albumin-to-creatinine ratio to assess the target-organ damage; LVMI was utilized to evaluate the cardiac organ damage, and the urinary albumin-to-creatinine ratio was a surrogate marker for renal organ damage; in their results, they found urinary albumin-to-creatinine ratio was associated with beat-to-beat DBPV significantly; however, neither SBPV or DBPV was correlated with LVMI remarkably. However, there were some drawbacks of those studies. Parati *et al*.^30^ only used SD of BP values to evaluate the BPV, and the study subject of Veerman *et al*.^31^ was quite small. Furthermore, Wei *et al*.^6^ also explored the correlation between beat-to-beat SBPV and target-organ damage; they measured 10 minutes beat-to-beat BP in 256 untreated hypertensive subjects with Finometer device; they also measured LVMI, urinary albumin-to-creatinine ratio and pulse wave velocity (PWV) to assess the target-organ damage; PWV was used to estimate the arterial stiffness (and its inverse, arterial compliance); ARV, VIM and the difference between maximum and minimum BP (MMD) were used to estimated the BPV; and then they demonstrated that LVMI increased with the three indices of SBPV, and the urinary albumin-to-creatinine ratio only increased with MMD of SBP, while, none of the three indices of SBPV was associated with PWV significantly. In our study, we used TAC to evaluate the vascular elasticity and utilized SD, ARV, RSD and VIM of beat-to-beat BP values to assess the beat-to-beat BPV. And we demonstrated that all of the four indices of SBPV were associated with vascular elasticity; in addition, SD, RSD and VIM of DBP were also correlated with TAC in bivariate correlation analysis; whereas, in multivariate linear regression analysis, only VIM of SBP was associated with TAC independent of SBP, DBP, age and BMI in hypertensive population.

Our results also provided the methodology to quantify the BPV and its prognostic significance. It was crucial to use proper parameters to assess BPV. Some previous studies used different parameters to evaluate the BPV, and found the BPV were controversial to predict risk of CVD^6, 22, 30–34^. SD mainly reflected the dispersion of BP measurements around the mean value without taking accounting of the time series of the BP recordings^7^; moreover, as SD mainly reflected the dispersion of BP recordings around the mean value, it could be influenced by the mean BP^9^. To overcome these deficiencies of the SD, we used ARV, RSD and VIM besides SD to estimate BPV in this study. As the ARV was calculated by the average level of absolute differences of consecutive measurements, it took the time series of BP measurements into account and it was also less sensitive to relatively low frequency sampling of non-invasive monitoring^7^. RSD was the square root of the total squared differences of data points from a linear regression of blood pressure values against time^8^. When the BP fluctuation had an underlying trend over time, especially the relationship between BP and time was approximately linear, RSD was more suitable than SD to estimate the extent of variability. VIM was a transformation of SD that was defined to be uncorrelated with mean levels for all individuals in the cohort^9^. Using VIM as an index of variability might help to overcome some of the difficulties associated with mean BP levels. In other words, the VIM could eliminate the confounding interference of the mean BP values. Indeed, in multivariate linear regression analysis, our result authentically showed that the VIM of SBP was correlated with TAC independent of SBP values in hypertensive population.

In addition, some other interesting results in this study deserve discussion. Our results showed that SBP and all of the indices of SBPV in the hypertensive population were larger than the values in the normal population significantly. However, DBP and all the indices of DBPV in the hypertensive population were not significantly different from the values in the normal population. Similarly, HR parameters in the hypertensive population were also not significantly different from the values in the normal population. Furthermore, the TAC in the hypertensive subjects was smaller than the values in the normal subjects remarkably. And the closer correlation was found between SBPV and TAC. Schillaci *et al*.^2^ found that arterial stiffness together with stroke volume determined the enhancement of pulse pressure; in return, increased BP evoked a passive augment of in arterial stiffness through the recruitment of collagen fibers in the wall. And the TAC was the inverse of arterial stiffness. These explained that the correlation of TAC with SPBV was closer than DBPV.

Notwithstanding the novelty of our results, we should interpret the limitations of our study. In this study, SV/PP was used to assess TAC. This method was based on a three-element Windkessel model^14–16, 26, 29^. In the three-element Windkessel model, it assumed that the heart follows Poiseuille’s Law and the ratio of pressure to volume in the chamber was constant^14^. When the structural changes existed in some disease states, this presumption might not be valid. However, the addition of more elements evoked the increase in variance, but could not improve the accuracy. In addition, the calculation of SV/PP was used peripheral PP instead of central PP to estimate the TAC^15^. In patients with no or minor amplification of pulse pressure from aorta to periphery, this approximation could be accurate; nevertheless, for patients with subjects exhibiting physiological pulse wave amplification, it might underestimate the TAC. To improve the sensitivity and specificity of TAC estimation, the definition of TAC should have a consensus. Moreover, Heitmar *et al*.^14^ suggested that some additional parameters, such as arterial pulse pressure waveform, be added to analyze the TAC.

In addition, this study was a cross-sectional study. Considering the cross-sectional nature of the present study, we could not deduce the existence of any causal link between beat-to-beat BPV and TAC. In other words, increased beat-to-beat BPV might evoke the reduction of the TAC or versa. Larger longitudinal studies were needed to evaluate the prognostic implications of beat-to-beat BPV on TAC in a hypertensive population. Furthermore, the sample size of the current study was relatively small. The relatively small sample size might have some influences on the results. Lastly, the age range of the hypertensive population and normal population was relatively large, and it might also affect the accuracy of the results.

## Conclusion

In the present study, we studied the influence of beat-to-beat BP parameters on the vascular elasticity in hypertensive population. Our results showed that the beat-to-beat BPV, especially the beat-to-beat SBPV, was an independent determinant of vascular elasticity. As the reduced vascular elasticity respected the alterations of structural and functional vascular properties, the beat-to-beat BPV might have an impact on structural and functional vascular properties in hypertensive population.

## Electronic supplementary material

Dataset 1

Dataset 2
